# Perioperative and Intensive Care Management of Pediatric Tracheal Tear

**DOI:** 10.1155/2014/738216

**Published:** 2014-02-19

**Authors:** Sanjay M. Bhananker, Ramesh Ramaiah

**Affiliations:** Department of Anesthesiology and Pain Medicine, Harborview Medical Center, P.O. Box 359724, 325 9th Avenue, Seattle, WA 98104, USA

## Abstract

Management of tracheal tears can prove to be challenging in the perioperative setting. This is a rare condition that can be life threatening. Here, we present a case of seven-year-old boy involved in a high-speed motor vehicle collision. The child sustained multiple injuries including a near fatal head injury, multiple facial fractures, and a tracheal injury associated with pneumomediastinum. Due to the imminent threat of brainstem herniation while being imaged in the CT scanner, the patient underwent an emergent craniotomy to evacuate his evolving intracranial bleed. Imaging prior to the craniectomy suggested a possible tracheal injury, given the extensive pneumomediastinum. However, initial perioperative ventilation was without any difficulty. After stabilization of intracranial pressure (ICP) and hemodynamics, on hospital day 4, the patient returned to the operating room to diagnose and repair his tracheobronchial injury. This is a unique polytrauma case in which a tracheal tear was managed in the midst of other life-threatening injuries.

## 1. Introduction

Tracheal bronchial injuries, whether iatrogenic or traumatic, can be challenging to manage perioperatively due to challenges in securing the airway, ventilation difficulty, and hemodynamic instability [1]. Traumatic tracheobronchial injuries may be underreported secondary to their association with high mortality prior to operative repair [2]. Furthermore, reports of spontaneous tracheal injury are even rarer [3].

In this report, we present this interesting case in which the mechanism of injury was likely to be consistent with a possible burst fracture type of phenomenon where increased intrathoracic pressure occurred against a closed glottis at the time of injury.

## 2. Case History

The patient was a seven-year-old, 22 kg boy who was a backseat, restrained passenger involved in a high-speed motor vehicle collision. He sustained severe head trauma with a depressed frontal skull fracture and subdural hematoma. He was intubated in the field by the paramedics, with a 6.0 cuffed ET tube. He was taken emergently to the operating room for a decompressive craniectomy after he was noted to be unstable in the CT Scanner due to an imminent brainstem herniation. In the preoperative scans, there was evidence of air tracking from the right mediastinum up to the right common carotid artery, continuing up to the C2-C3 disk level.

The patient underwent a bilateral frontal and right temporal craniectomy. A 6.0 cuffed ETT was *in situ* and secured at 15.5 cm at the level of the teeth. The patient was maintained on isoflurane, paralyzed with 15 mg rocuronium, and given a total of 60 mcg of fentanyl for analgesia. Ventilation parameters were set with the following volume control settings: PEEP of 2 cm H_2_O, tidal volume 250 mL, and respiratory rate 25. At these settings, peak pressure ranged from 17 to 27 cm H_2_O. Since preoperative imaging suggested the possibility of a tracheal injury, close attention was given to airway pressures. Equipment and personnel for insertion of chest drains were kept readily available, should the clinical condition deteriorate. Manual ventilation was notably easy both prior to incision and upon the end of the case: visual observation showed bilateral chest rise and auscultation was reassuring for bilateral breath sounds.

**Figure 1:**
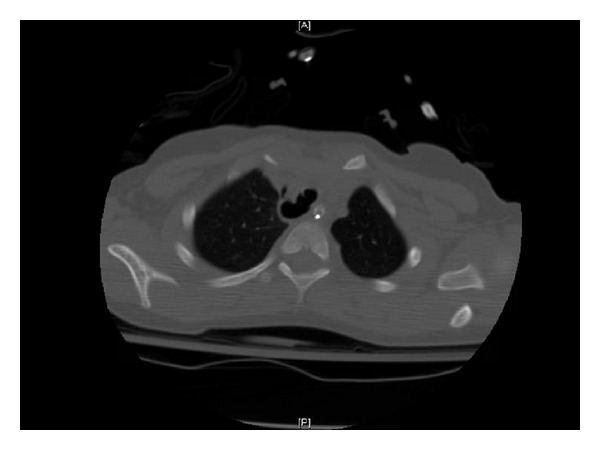
Hospital Day number 2. Noncontrast CT scan of chest. Extensive pneumomediastinum has diminished. There is a persistent right posterolateral tracheal wall defect/tracheal diverticulum.

He was kept intubated in ICU with similar ventilator parameters. CT scan of chest on the next day revealed minimal pneumomediastinum and a right posterolateral tracheal wall defect/tracheal diverticulum. (Figure 1) Tracheal suction with a suction catheter down the ET tube was undertaken so that the tip of the suction catheter was never beyond the tip of the ET tube (to avoid further trauma to the trachea).

**Figure 2:**
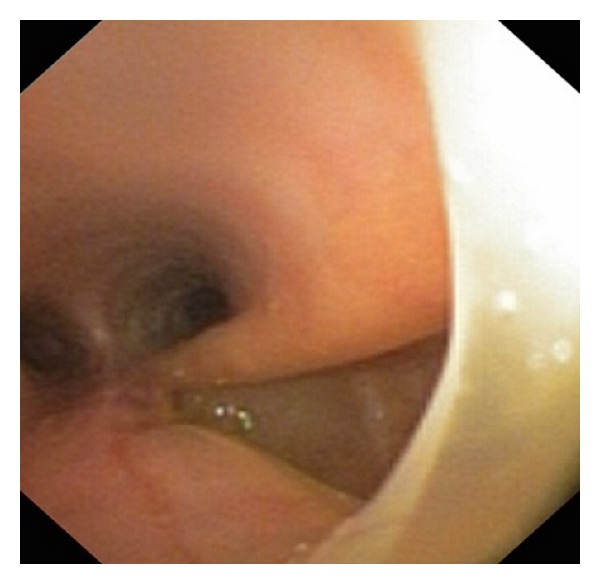
Prerepair bronchoscopic view of the tracheal tear.

On hospital day 4, the patient was cleared from the neurological standpoint to proceed to surgery for his tracheal repair. Again, the child had an *in situ* 6.0 cuffed ETT. Vascular access consisted of a right femoral central line, radial arterial line, and peripheral IVs. Induction was initiated with sevoflurane and patient was subsequently paralyzed with rocuronium 5 mg. A bronchoscopic examination of the tracheobronchial tree was performed by the attending thoracic surgeon, which revealed the tracheal tear approximately 3.5 to 4 cm in length, at the right membranocartilaginous junction (Figures 2 and 4). Thereafter, an endobronchial blocker was placed in the right main bronchus under direct bronchoscopic visualization.

A thoracic epidural was placed at T5-T6 for postoperative pain control. He was placed in a left lateral decubitus position; left lung ventilation begun and was deemed adequate for a right sided thoracotomy.

**Figure 3:**
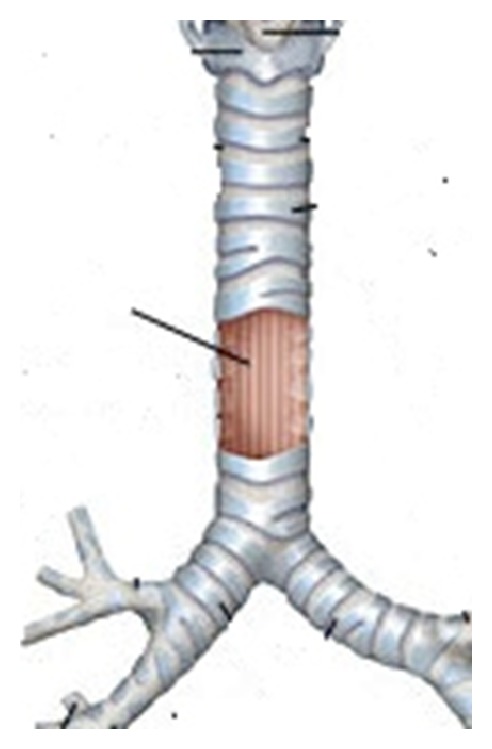
Location of the tracheal tear in the distal trachea.

**Figure 4:**
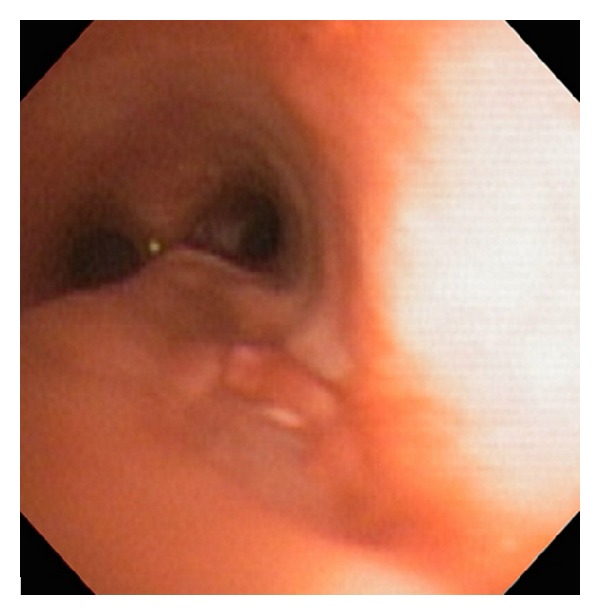
Postrepair bronchoscopic view of the tracheal tear.

The tracheal lesion was successfully repaired with a bovine pericardial patch (Figure 3). The patient did not meet extubation criteria at the end of the case due to his high respiratory rate. He was taken intubated and sedated in a stable condition to the pediatric intensive care unit. Four days later, the patient was successfully extubated. He underwent uneventful anesthetic including tracheal intubation for the repair of his facial fractures on postoperative day 9. He was discharged from the hospital on day 20 and transferred to rehabilitation for further care.

## 3. Discussion

Pediatric tracheobronchial lesions secondary to blunt injury are rare with mortality rates as high as 30%, and death likely occurring within an hour of the traumatic injury [4]. External forces produced by blunt trauma transmit directly onto the mediastinum, and given a child's pliable chest wall, can result in fatal disruptions of both the airway and major arterial vessels [5].

In this case, the 7-year-old boy sustained a head injury and facial fractures. The patient's clinical presentation was also significant for a possible tracheal injury suggested by CT imaging which has a 85% sensitivity of detecting airway lesions [6]. Common findings include pneumomediastinum without pneumothorax and deep cervical air and would have prompted further evaluation with bronchoscopy [6, 7]. However, his brain injury posed an immediate threat to his life and required urgent decompression.

The mechanism of injury was likely a possible burst fracture type of phenomenon where increased intrathoracic pressure occurred against a closed glottis at the time of injury. It is also possible that this injury occurred during intubation in the field from laceration by a protruding stylet. The tracheal tear could also have resulted from overinflation of the cuff, as a 6.0 cuffed tracheal tube was relatively large for this boy.

Given the stability of the patient during his initial surgery (stable peak pressures and no signs of pneumothorax), it was theorized that the endotracheal tube was likely below the tracheal lesion, and positive pressure ventilation was delivered for 4 days without complication. Bronchoscopy to diagnose or rule out tracheal injury was delayed until the patient's neurological status had improved.

The lesion measured approximately 3.5 to 4 cm in length, at the right membranocartilaginous junction, above the carina and just proximal to the entrance of the right mainstem bronchus. Right-sided bronchial injuries in the setting of chest trauma occur more frequently than on the left [4] and especially at this weak histological junction.

Surgical repair with right thoracotomy was performed due to size of lesion. Primary anesthetic management would be to secure the airway with endotracheal intubation with care not to exacerbate the injury. Fiberoptic bronchoscopy should be used to facilitate this. We placed the endotracheal tube distal to the lesion to facilitate positive pressure ventilation without creating tracheal perforation or rupture in this weakened part. Lack of double lumen tubes small enough for this particular patient at our institution also posed another added source of difficulty. Instead, a bronchial blocker was used. The bronchial blocker offered the advantage of allowing us to leave the *in situ* airway in place and provided adequate surgical exposure.

## 4. Conclusion

Tracheal tears in the pediatric population are rare and have the potential of being difficult to manage. The imminent threat of brainstem herniation in this 7-year-old polytrauma victim prompted immediate surgical decompression despite concerns for tracheal injury. With the diagnostic assistance of the CT Scan, the anesthesiologists were acutely aware of possible ventilation difficulties associated with a tracheal injury. While the patient was ventilated in the ICU for 4 days with the tip of the tracheal tube placed distal to the lesion, the perioperative management included one lung ventilation with a bronchial blocker and a throacic epidural for postoperative analgesia.
